# CREBH Regulates Systemic Glucose and Lipid Metabolism

**DOI:** 10.3390/ijms19051396

**Published:** 2018-05-08

**Authors:** Yoshimi Nakagawa, Hitoshi Shimano

**Affiliations:** 1 Department of Internal Medicine (Endocrinology and Metabolism), Faculty of Medicine, University of Tsukuba, Tsukuba, Ibaraki 305-8575, Japan; 2 International Institute for Integrative Sleep Medicine (WPI-IIIS), University of Tsukuba, Tsukuba, Ibaraki 305-8575, Japan; 3 Life Science Center, Tsukuba Advanced Research Alliance (TARA), University of Tsukuba, Tsukuba, Ibaraki 305-8577, Japan; 4 Japan Agency for Medical Research and Development–Core Research for Evolutional Science and Technology (AMED-CREST), Chiyoda-ku, Tokyo 100-1004, Japan

**Keywords:** CREBH, SREBP, LXRα, PPARα, lipid metabolism, transcription, FGF21

## Abstract

The cyclic adenosine monophosphate (cAMP)-responsive element-binding protein H (CREBH, encoded by CREB3L3) is a membrane-bound transcriptional factor that primarily localizes in the liver and small intestine. CREBH governs triglyceride metabolism in the liver, which mediates the changes in gene expression governing fatty acid oxidation, ketogenesis, and apolipoproteins related to lipoprotein lipase (LPL) activation. CREBH in the small intestine reduces cholesterol transporter gene *Npc1l1* and suppresses cholesterol absorption from diet. A deficiency of CREBH in mice leads to severe hypertriglyceridemia, fatty liver, and atherosclerosis. CREBH, in synergy with peroxisome proliferator-activated receptor α (PPARα), has a crucial role in upregulating *Fgf21* expression, which is implicated in metabolic homeostasis including glucose and lipid metabolism. CREBH binds to and functions as a co-activator for both PPARα and liver X receptor alpha (LXRα) in regulating gene expression of lipid metabolism. Therefore, CREBH has a crucial role in glucose and lipid metabolism in the liver and small intestine.

## 1. Introduction

Obesity is a high-risk metabolic disorder leading to various complications, including cardiovascular disease, hyperlipidemia, and type II diabetes [1,2,3]. Numerous cellular stress and inflammatory signaling pathways are activated by ectopic accumulation of fat in various tissues, resulting in insulin resistance, pancreatic β-cell dysfunction, and hepatic steatosis [4]. The liver is the central metabolic organ regulating the key aspects of glucose and lipid metabolism, including gluconeogenesis, fatty acid β-oxidation, lipoprotein uptake and secretion, and lipogenesis [5]. Given that the portal vein is the critical path along which insulin signaling is conveyed from the pancreas during the fed state, the hepatic glucose and lipid metabolism are directly under the control of nutrient signaling.

Glucose and lipid metabolism are regulated by cooperating transcription factors. cAMP response element-binding protein (CREB) is a typical transcriptional factor that regulates gluconeogenic gene expression in an energy-depleted condition. A typical transcription factor for lipid metabolism is the membrane-bound protein, sterol regulatory element-binding protein (SREBP). The three isoforms of SREBPs are SREBP-1a, SREBP-1c, and SREBP2, which localize in the endoplasmic reticulum (ER). SREBP-1c mainly regulates fatty acid synthesis gene expression. SREBP-2 regulates cholesterol synthesis gene expression. SREBPs are escorted by SREBP cleavage activation protein (SCAP), a cholesterol sensor protein, to Golgi, thereby cleaved by site-1 protease and site-2 protease, and transferred to the nucleus. SREBPs thus play a pivotal role in lipid metabolism.

However, despite numerous studies, the mechanisms of transcription in metabolism do not fully remain clear. Therefore, we need to better understand the functions of transcription factors in regulating gene expression including the metabolism of glucose, triglyceride, and cholesterol. Cyclic AMP-responsive element-binding protein 3-like 3 (CREB3L3, CREBH) possesses similarity to SREBP with regards to its localization and the activation process of its cleavage system [6]. CREBH has a homology with the cAMP response elements (CRE)/activating transcription factor (ATF) family molecules and binds to the same consensus sequences as these molecules [6]. Consistent with this, CREBH also increases gluconeogenesis-related gene expression. In contrast, CREBH can activate hepatic expression of *Fgf21*, an anti-metabolic syndrome hormone [7,8]. Mutations in CREBH have been identified in patients with extreme hypertriglyceridemia, and these mutations produce no functional CREBH protein. CREBH has a crucial role in triglyceride (TG) metabolism to regulate the expression of apolipoproteins related to lipoprotein lipase (LPL) activation in the liver [9]. More intriguingly, SREBP and CREBH make a good contrast for activation in nutritional abundance and depletion, respectively. CREBH might antagonize SREBP functions, leading to an improvement in lipid metabolism. This review summarizes the new transcriptional factor CREBH, which controls glucose and lipid metabolic genes (see Table 1).

## 2. The Gene Regulation of CREB3L3 in Response to Nutrient Condition

The liver-specific transcription factor CREBH is a basic leucine zipper domain member of the CREB/ATF family. The amino acid sequence of CREBH contains a region extensively homologous to the b-Zip domain for three transcription factors belonging to the CREB/ATF family: Drosophila box-B binding factor-2 (BBF-2), human leucine zipper protein (LZIP), and mouse old astrocyte specifically induced substance (OASIS). Between the b-Zip domain and the other leucine zipper, CREBH also contains a hydrophobic stretch of 17 amino acids that may potentially constitute a transmembrane domain similar to that found in LZIP [6]. The KDEL-like sequence in CREBH—“GDEL”—can behave as an ER retrieval sequence. Within the putative transmembrane domains and a portion of the lumenal domains of regulated intramembrane proteolysis (RIP)-regulated ER-localized proteins, CREBH displays a high degree of sequence conservation. Homologous sequences of the cleavage by site-1 protease (S1P) and site-2 protease (S2P) of the SREBP and the activating transcription factor 6 (ATF6) are found in CREBH. Located in the ER, CREBH contains a transmembrane domain homologous to those of SREBP and ATF6. Under ER stress, CREBH moves to the Golgi apparatus, where S1P and S2P cleave its amino-terminal portion. The amino-terminal portion of CREBH transfers to the nucleus, inducing genes responsible for the systemic inflammatory response [15]. Fasted or insulin-resistant states induce *CrebH* expression, resulting in the accumulation of the nuclear form of CREBH [21]. In fasted states, glucagon-protein kinase A (PKA) signaling activates *CrebH* expression and then activates CREBH transcriptional activity via post-translational modification [11]. Glucocorticoids produced and secreted by the adrenal gland bind to hepatic glucocorticoid receptors (GRs), which exert antagonizing effects on insulin and promote gluconeogenesis. Activated GRs induce *CrebH* expression by directly binding to the glucocorticoid transcriptional response element in the promoter region of CREBH [11]. *CrebH* expression is also induced by some kinds of fatty acids such as palmitate, oleate, and eicosapenonate. via mediating PPARα activation [21]. Thus, *CrebH* expression in the liver is efficiently increased by PPARα agonists such as fenofibrate, Wy14643, and pemafibrate [10,21,22]. In fact, *CrebH* promoter contains a peroxisome proliferator responsive element (PPRE) for PPARα transactivation [21]. In the livers of PPARα KO mice, *CrebH* expression is significantly reduced; conversely, in the livers of CREBH KO mice, *Ppara* expression is significantly decreased [10]. *Ppara* promoter also contains a CREBH binding site (CHRE) [10]. PPARα agonist-mediated gene expression requires CREBH because it is suppressed in CREBH KO mice [10]. CREBH and PPARα form mutual auto-loop regulation at the transcription level. In the liver, but not in the intestine, hepatocyte nuclear factor 4α (HNF4α)—a transcription factor for gluconeogenesis—directly binds to the promoter of *CrebH* and activates its expression [23]. In the refed state, *CrebH* expression is suppressed by insulin [24].

*CrebH* expression is significantly induced by proinflammatory cytokines such as interleukin 6 (IL6), IL1β, and tumor necrosis factor α (TNFα), as well as ER stress inducers such as dithiothreitol (DTT), thapsigargin, and Brefeldin A (BFA) [15]. CREBH interacts with activating transcription factor 6 (ATF6)—an ER stress-related transcription factor—to synergistically activate gene expression of major acute phase response (APR) genes such as serum amyloid P-component and C-reactive protein [15]. However, there seems a controversy about the induction of *CrebH* expression in response to ER stress [14]. Further investigation into CREBH and ER stress especially in relation to ATF6 is necessary.

## 3. CREBH Regulates *Fgf21* Expression in the Liver and Subsequently Regulates Glucose and Lipid Metabolism 

**Figure 1:**
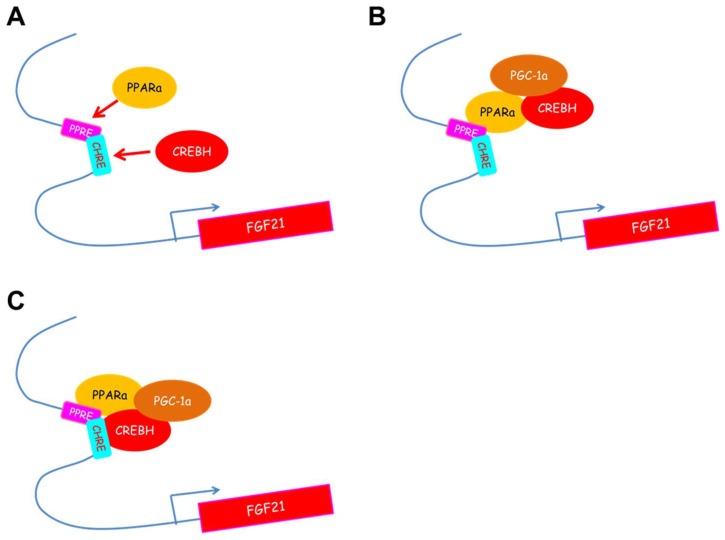
Scheme of *Fgf21* transcriptional regulation by the interaction between CREBH and PPARα. *Fgf21* promoter has a PPARα-binding site (PPRE) and a CREBH-binding site (CHRE), which are partially overlapped. (**A**) CREBH and PPARα directly bind to their own binding sites on the *Fgf21* promoter. (**B**) CREBH regulates PPARα transcriptional activity as a co-activator and by peroxisome proliferator-activated receptor gamma coactivator 1α (PGC-1α) recruiting. (**C**) CREBH and PPARα form a complex on the *Fgf21* promoter and synergistically activate *Fgf21* expression.

CREBH directly binds to the proximal region of the *Fgf21* promoter and upregulates *Fgf21* expression. Overexpression of CREBH in the liver upregulates hepatic *Fgf21* expression, accompanied by an increase in plasma levels of fibroblast growth factor 21 (FGF21), a unique member of the FGF family with hormone-like actions [25]. FGF21 is a key mediator of starvation that activates lipolysis in white adipose tissue (WAT) and increases fatty acid oxidation and ketogenesis in the liver [26,27] and has therapeutic effects on obesity-related metabolic disturbances such as insulin resistance, diabetes, and hypertriglyceridemia in ob/ob mice, diet-induced obese mice, and diabetic monkeys [28,29]. *Fgf21* expression is well known to be regulated by PPARα, which plays a key role in lipid oxidation and is induced by fasting or by consumption of a ketogenic diet (high-fat, low-carbohydrate diet) [26,27]. In a fasted state and fed on a ketogenic diet, CREBH KO mice markedly suppress *Ppara* and *Fgf21* expression [7,10]. Cooperation between CREBH and PPARα upregulates *Fgf21* expression [8,10]; the two operate as transcriptional co-activators [8]. Nuclear CREBH activates the *Ppara* promoter in an autoloop fashion and is crucial for the ligand transactivation of PPARα by interacting with its transcriptional regulator, peroxisome proliferator-activated receptor gamma coactivator-1α (PGC-1α) (Figure 1) [10]. Consequently, the target genes of CREBH and PPARα are overlapped. In comparisons between CREBH KO mice and PPARα KO mice in a fasted condition and fed a ketogenic diet, the direct targets of CREBH are identified as *Cpt1a*, fatty acid oxidation, and *Bdh1*, ketogenesis [7]. Both CREBH and PPARα are crucial transcription factors in fatty acid oxidation and ketogenesis in the livers of energy-depleted mice. 

The overexpression of the active portion of CREBH in the livers of mice ameliorates the physiology of diet-induced obesity, hypertriglyceridemia, hyperglycemia, insulin resistance, and obesity. CREBH significantly induces *Ppara* and its target genes—including fatty acid oxidation genes such as *Acox1* and *Cpt1a*—indicating that CREBH can activate fatty acid oxidation in the liver. CREBH regulates the gene expression of lipoprotein lipase modulators such as *Apoa4*, *Apoa5*, *Apoc2*, and *Apoc3*, resulting in the activation of LPL activity [9]. The increase in plasma FGF21 levels caused by CREBH overexpression leads to increased energy expenditure with the increase of thermogenesis genes such as *Ucp1* and *Ppargc1a* in WAT [10].

In contrast, some reports have revealed that *Fgf21* expression is regulated via various transcription factors (Table 2). Endoplasmic reticulum (ER) stress regulates *Fgf21* expression via ER stress-related transcription factors, including activating transcription factor 4 (ATF4), CCAAT enhancer binding protein homologous protein (CHOP), and X-box-binding protein 1 (XBP1) [30,31,32]. In response to amino acid deprivation, ATF4 induces *Fgf21* expression [32]. Mitochondrial dysfunction induced by autophagy deficiency in the skeletal muscle increases *Fgf21* expression depending on ATF4 [33]. XBP1 has an indirect effect on *Fgf21* expression. XBP1 directly activates *Ppara* expression and subsequently increases *Fgf21* expression in a fasting condition [34]. In an overnutrient condition, *Fgf21* expression is also increased. It depends on the carbohydrate-responsive element-binding protein (ChREBP), which is efficiently activated by carbohydrates, including glucose and fructose [35]. ChREBP-mediated *Fgf21* expression requires PPARα, inducing the accessibility of ChREBP to the carbohydrate-responsive element (ChoRE) site in the *Fgf21* promoter [36]. Conversely, liver X receptor (LXR) downregulated *Fgf21* expression in the liver when fed with a high-cholesterol diet [37].

## 4. CREBH Regulates Gluconeogenesis Gene Expression

CREBH can bind to both CREs and Box B sequences. CREs are the response elements for CREB, which contains gluconeogenesis genes such as phosphoenolpyruvate carboxykinase 1, cytosolic (*Pck1*), glucose-6-phosphatase, and catalytic (*G6pc*). These genes are upregulated in the livers of mice in a fasted state via directly binding CREB to CRE sequences in the promoter region of these genes. Conceivably, CREBH was also reported to bind to and upregulate these genes [11,12]. CREBH co-operates with CREB/CREB-regulated transcriptional coactivator 2 (CRTC2), a CREB transcriptional modulator, to activate *Pck1* and *G6pc* expression [11]. During fasting or in the insulin-resistant state, *CrebH* expression is induced by the glucocorticoid receptor (GR)/PGC-1α complex, and the HNF4α/PGC1α complex [11]. CREBH also regulates the rate-limiting enzymes for glycogenolysis liver glycogen phosphorylase (*Pygl*) expression [38]. It has been reported that adenoviral CREBH overexpression in the livers of mice induces gluconeogenesis genes and subsequently increases plasma glucose levels while adenoviral knockdown of CREBH in the livers of mice significantly reduces blood glucose levels [11]. In contrast, our report shows that although liver CREBH in transgenic mice and adenoviral CREBH overexpression in the livers of mice induce gluconeogenesis genes, both the hepatic expression and the plasma levels of FGF21 are significantly increased in these mice, resulting in decreased plasma glucose levels [10]. The effects of CREBH on the regulation of plasma glucose could be context-dependent. 

## 5. CREBH Regulates Lipid Metabolism in Fatty Liver

### 5.1. Deficiency of CREBH Exacerbates Non-Alcoholic Fatty Liver Disease (NAFLD) and Non-Alcoholic Steatohepatitis (NASH)

CREBH KO mice, when fed an atherogenic high-fat (AHF) diet, show a massive accumulation of hepatic lipid metabolites and a significant increase in plasma TG levels. CREBH KO mice increase Non-Alcoholic Steatohepatitis (NASH) activities. In this metabolic stress, CREBH increases gene expression related to (1) triglyceride synthesis: FA synthase (*Fasn*), acetyl co-enzyme A (CoA) carboxylase 1 (*Acc1*), *Acc2*, Stearoyl-CoA desaturase 1 (*Scd1*), and diacylglycerol acetyltransferase 2 (*Dgat2*); (2) cholesterol synthesis: 24-dehydrocholesterol reductase (*Dhcr24*) and long-chain-FA-CoA ligase 1 (*Acs1*); (3) fatty acid elongation: elongation of very-long-chain FAs protein (*Elovl*)*2*, *Elovl5*, *Elovl6*, and peroxisomal trans-2-enoyl-CoA reductase (*Pecr*); (4) fatty acid oxidation: *Cpt1a*, *Cyp4a10*, *Cyp4a14*, *Cyp2b9*, *Cyp2b13*, FA desaturase (*Fads*)*1*, *Fads2*, *Acox1*, and *Ppara*; (5) lipolysis: *Apoc2*, *Apoa4*, *Apoa5*, and *Apoc3*; (6) lipolysis-stimulated lipoprotein receptor: lecithin-cholesterolacyl transferase (*Lcat*), and acyl-CoA thioesterase *4* (*Acot4*); and (7) lipid transport: sterol carrier protein 2 (*Scp2*) [18]. The upstream genes for lipogenic regulators, including *Chrebp*, *Lxra*, PPARγ-coactivator-1α (*Ppargc1a*), *Ppargc1b*, and fat-specific protein 27 (*Fsp27*), are controlled by CREBH. Lipid droplet growth and TG storage in white adipocytes is promoted by FSP27, a lipid droplet-associated protein. There are two FSP27 isoforms, namely, FSP27α and FSP27β. FSP27β contains 10 additional amino acids at the N-terminus of the original FSP27 (FSP27α). WAT and the liver specifically express *Fsp27α* and *Fsp27β* transcripts, respectively, which are driven by distinct promoters. The *Fsp27β* promoter is directly activated by CREBH [19]. Using a common NASH model—methionine choline-deficient (MCD) diet feeding—the effects of CREBH on NASH were evaluated. CREBH tissue-specific KO mice were developed using the one-step clustered regularly interspaced short palindromic repeats/CRISPR-associated endonuclease 9 (CRISPR/Cas9) system [39]. Liver-specific CREBH KO mice also displayed severe hepatitis in MCD diet feeding without an increase in liver lipid contents [39]. The plasma marker levels for liver injury—such as alanine transaminase (ALT) and aspartate transaminase (AST)—are severely increased by a deficiency of CREBH in the liver, which also significantly increases the gene expression of inflammation and liver fibrosis [39]. The deficiency of CREBH in the liver could have a crucial role in developing NAFLD and NASH. CREBH, activated by triglyceride accumulation, induces FGF21, which suppresses adipose tissue lipolysis, ameliorating hepatic steatosis [40]. When fasted or fed a ketogenic diet, CREBH KO mice develop severe hepatic steatosis because of decreased hepatic fatty acid oxidation [7] and increased adipose tissue lipolysis [40]. A ketogenic diet activates both *CrebH* and *Fgf21* expression, indicating a positive correlation between both factors [7]. FGF21 production was impaired in CREBH KO mice, and adenoviral overexpression of FGF21 suppressed adipose tissue lipolysis and improved hepatic steatosis in these mice [40]. In a negative feedback loop, CREBH regulates non esterified fatty acids (NEFA) flux from adipose tissue to the liver via FGF21. Supporting the role of CREBH in lipogenesis and lipolysis, the overexpression of the activated form of CREBH protein in the liver significantly increases the accumulation of hepatic lipids but reduces plasma TG levels in mice [40]. Taken together, the better strategy for improving fatty liver and hyperlipidemia—CREBH overexpression or CREBH deficiency—remains unclear.

A cluster of ER membrane-bound proteins, including insulin-induced gene-1 (Insig-1) and gene-2 (Insig-2), and SREBP cleavage-activating protein (SCAP), control the regulation of SREBP signaling [41,42,43]. CREBH induces the expression of a liver-specific isoform of Insig-2—*Insig-2a*—which downregulates the translocation of SREBP-1c from the ER to the Golgi and reduces de novo lipogenesis [17]. The CREBH-Insig-2a signaling pathway inhibits hepatic de novo lipogenesis and prevents the onset of hepatic steatosis and hypertriglyceridemia [17]. CREBH and SREBPs interact to regulate lipid metabolism. CREBH is activated by energy shortage; conversely, SREBPs are activated by overnutrition. These two molecules keep a balance to maintain cellular lipid levels at the transcriptional level. CREBH is therefore a key metabolic regulator of hepatic lipogenesis, fatty acid oxidation, and lipolysis under metabolic stress [18].

### 5.2. CREBH Regulates Very Low-density Lipoprotein( VLDL) Particle Metabolism in Fatty Liver

Liver TG content is regulated by the balance between fatty acid uptake, synthesis, and oxidation, and TG synthesis and export via secretion of TG-rich VLDL [44,45]. In NAFLD, both TG synthesis and secretion are increased, but TG export is insufficient to prevent steatosis [46,47]. The assembly of a greater number of VLDL particles and/or larger VLDL particles containing more core TG increases TG export in the liver as well as VLDL secretion [48,49]. Plasma VLDL levels increase during APR, but ApoB, a molecule constituting VLDL, is not clarified as the APR gene [50,51]. CREBH is reported to activate *ApoB* expression [15]. *ApoB* expression is reduced in the fetal livers of CREBH KO mice, and CREBH binds to the *ApoB* promoter region, resulting in an increased *ApoB* expression [15,16]. TG-rich lipoprotein secretion is upregulated in wild-type (WT) mice treated with an acute fat load, but this phenomenon is not observed in CREBH KO mice [16]. TNFα treatment activates *CrebH* expression and increases ApoB biosynthesis and VLDL secretion in the liver [16]. Lipopolysaccharide (LPS)- or high-fat diet-induced inflammation also increases ApoB production, resulting in hyperlipoproteinemia in WT mice but not in CREBH KO mice [16]. It is possible that CREBH could mediate inflammation and hepatic VLDL overproduction in chronic metabolic diseases.

Apoa4 increases after TG absorption to facilitate intestinal chylomicron assembly and TG secretion [52,53]. Hepatic *Apoa4* expression is increased with high hepatic TG levels in steatosis [14] and both acute and chronic hepatosteatosis [54]. CREBH is identified as a major regulator responsible for *Apoa4* expression in both the liver and the intestine [14].

Apoa4 affects the trafficking kinetics of nascent ApoB-containing lipoproteins through a direct association with ApoB in the secretory pathway [55,56]. Apoa4 also regulates hepatic lipid content by activating nascent VLDL particle expansion and TG efflux without increasing the number of ApoB-containing lipoprotein particles secreted from the liver [54]. The direct interaction between Apoa4 and the amino terminus of ApoB slows the secretory trafficking of VLDL particles, allowing the addition of more lipid molecules to the expanding VLDL particle before secretion [56]. Thus, Apoa4 plays a crucial role in VLDL particle expansion during TG-rich lipoprotein assembly and in mobilizing TG for secretion, which protects against hepatosteatosis without increasing the demand for ApoB synthesis. The reduction of ApoB or microsomal triglyceride-transfer protein (MTP) activity attenuates VLDL particle assembly, which attenuates CREBH processing and *Apoa4* expression, despite a dramatic increase in liver TG content.

Increasing hepatic TG content is necessary, but not sufficient, for CREBH-dependent Apoa4 activation. Instead, an aspect of the VLDL assembly and secretion pathway is essential for CREBH activation. Hepatocytes are unique relative to the non-lipoprotein-producing cells in that TG synthesis and storage must be coupled to their translocation across the ER membrane to form lumenal lipid droplets, which then serve as a substrate for TG acquisition by ApoB [49,57]. Steatosis-induced *Apoa4* expression leads to increased TG secretion and a reduction in hepatic lipid content by promoting VLDL particle expansion without increasing the number of VLDL particles [54]. This pathway probably evolved to increase hepatic TG flux from the steatotic liver into the plasma through VLDL particle expansion, thereby protecting the liver from lipid toxicity. 

Hepatic Apoa4 expression is strongly increased in the mouse models of steatosis [54,58]. Hepatosteatosis-induced hepatic *Apoa4* expression is regulated by the proteolytic processing of CREBH [14]. The fact that ApoB and MTP deficiencies block CREBH processing suggests that lipid movement into the ER, or another related function of these proteins, initiates vesicular trafficking of CREBH to the Golgi and CREBH processing to release the active form of CREBH. CREBH and Apoa4 play a coordinated role in promoting the assembly and secretion of larger, TG-enriched VLDL particles, thereby increasing hepatic TG efflux without increasing the number of VLDL particles. 

## 6. CREBH Regulates Lipoprotein Metabolism

### 6.1. CREBH Regulates the Expression of Apoa4, a Multitasking Apolipoprotein, in the Liver and Small Intestine

Apoa4, an apolipoprotein associated with high-density lipoproteins (HDLs) that is expressed and secreted in the liver and the small intestine, is a direct target for CREBH [14]. In mouse models, *Apoa4* expression in the liver strongly increases during steatosis [54,58,59]. In humans, *Apoa4* is primarily expressed in the small intestine [60,61]. Human genome-wide expression profiling studies have revealed that hepatic *Apoa4* expression is also induced during steatosis, and that both alcoholic and nonalcoholic steatohepatitic CREBH induction increases hepatic *Apoa4* expression. Conversely, research on CREBH KO mice reveals a reduction in *Apoa4* expression in both the liver and the small intestine [14]. 

Apoa4 is transferred from chylomicrons and VLDL to HDL in exchange for ApoCs, thereby activating lipolysis of TG-rich lipoproteins by LPL [62,63]. Apoa4 plays a role in reverse cholesterol transport and affords protection from atherosclerosis [64], and is also involved in fat absorption in the small intestine [65,66,67], the central regulation of food intake [68], and the regulation of insulin secretion from β-cells [69]. CREBH contributes to these Apoa4-mediated actions in maintaining the systemic and whole-body lipid metabolism. 

Leucine zipper protein (LZIP) is a CREBH-like transcription factor containing a transmembrane domain [70]. The DNA binding domain of LZIP shares 84% homology with that of CREBH [6]. LZIP regulates *Apoa4* expression because LZIP and CREBH share the promoter-binding region [71]. In addition, there is a possibility that LZIP and CREBH form a complex that mediates *Apoa4* expression [71].

### 6.2. CREBH Regulates Lipoprotein Metabolism in Response to Endotoxemia

Bacterial infections induce various physiological changes and inflammation as well as affect metabolism, particularly lipid metabolism in the host, which may result in hyperlipidemia [72]. During infections, an increase in lipoprotein production and dysfunction of circulatory lipoprotein clearance mechanisms cause TG levels to increase [73]. Plasma lipoproteins, particularly HDL, are markedly reduced in sepsis. Clinical studies reveal that low plasma HDL is a prognostic factor in severe sepsis [74,75], and HDLs may have a protective role in sepsis and endotoxemia as they decrease the levels of circulating LPS [76,77].

CREBH functions as a stress-responsive transcription factor [15,18]. In response to LPS, *CrebH* expression is upregulated in the liver [16,78]. TRAF6, an E3 ligase in the toll-like receptor (TLR) signaling pathway, is involved in the regulation of target molecules via ubiquitination [79]. TRAF6 is reported to be a crucial molecule in inflammation [56]. CREBH interacts with TRAF6, which induces CREBH cleavage and subsequent activation of its transcriptional activity via ubiquitination [78]. In response to LPS stimulation, CREBH is activated and then upregulates *Apoa4* expression and subsequently promotes the production of HDLs as a part of the host response to bacterial infection [78]. CREBH has a crucial role in endotoxin-triggered HDL production and protects the liver against endotoxin-induced injury [78]. 

## 7. Intestinal CREBH Overexpression Controls Intestinal Cholesterol Absorption

On feeding an AHF diet, mice overexpressing the active form of CREBH in the intestine exhibited an apparent reduction of gallstone formation in gall bladders and plasma cholesterol levels compared with those in WT mice [20]. CREBH increased cholesterol levels in feces and reduced intestinal cholesterol levels, thereby indicating that CREBH suppresses the absorption of cholesterol from the diet in the small intestine [20]. Niemann Pick C1-like 1 (NPC1L1) is a protein localized at the brush border membrane of the enterocytes, mediating cholesterol absorption into the enterocytes. Ezetimibe is a drug for hyperlipidemia that inhibits cholesterol absorption by blocking NPC1L1 intestinal transporters, resulting in a decrease in plasma cholesterol levels. CREBH reduces *Npc1l1* expression, leading to a reduction in cholesterol absorption from the small intestine and in plasma cholesterol levels [20]. CREBH might be a therapeutic target for the treatment of hyperlipidemia by inhibiting cholesterol absorption. However, a thorough understanding of the CREBH functions in the small intestine is currently lacking. Future studies in this area are necessary.

## 8. CREBH Regulates the Progression of Atherosclerosis

Atherogenic dyslipidemia with high plasma TG and LDL levels and low plasma HDL levels is a risk factor for atherosclerosis and cardiovascular disease (CVD). Patients with combined homozygous mutations in the glycosylphosphatidylinositol-anchored high-density lipoprotein–binding protein 1 (GPIHBP1) gene exhibit hypertriglyceridemia and severe CVD, suggesting that LPL-mediated TG clearance is involved in atherosclerosis [80]. Apoa1 is produced in the liver and small intestine and constitutes the predominant component of HDL [81]. Apoa1 interacts with the ATP-binding cassette transporter A1 (ABCA1) and activates cholesterol efflux from peripheral tissues for reverse cholesterol transport [82,83]. Apoa1 deficiency in low-density lipoprotein receptor (LDLR) KO mice increases non-HDL-C, thereby accelerating the process of atherosclerosis [84]. Hepatic *Apoa1* expression is reduced in CREBH KO mice and increased in primary mouse hepatocytes overexpressing CREBH, suggesting that CREBH might have a function in HDL metabolism [14]. CREBH deficiency suppressed *Apoa1* expression in both the liver and the intestine and reduced plasma Apoa1 and HDL-C levels, indicating that CREBH has a crucial role in the regulation of *Apoa1* expression [13]. HNF4α, which activates *CrebH* expression, also activates hepatic *Apoa1* expression [85,86,87,88]. Thus, CREBH and HNF4α co-operate to activate *Apoa1* expression [13]. Apoa4 is involved in HDL metabolism by activating lecithin:cholesterol acyltransferase, a key enzyme involved in the transfer of cholesterol to newly synthesized HDL particles via the conversion of free cholesterol into cholesteryl esters [89,90], which stimulates cholesterol efflux from macrophages [91] and activates the receptor-mediated uptake of HDL by hepatocytes [92].

Furthermore, transgenic overexpression of human or mouse Apoa4 conferred protection against atherosclerosis in mice [64,93,94]. CREBH deficiency results in high VLDL-TG and low HDL-C levels in the plasma and accelerated atherosclerosis in LDLR KO mice. In contrast, CREBH overexpression in the liver reduces plasma TG by activating LPL-mediated TG clearance by the transcriptional activation of apolipoprotein genes, such as *Apoa1*, *Apoa4*, *Apoa5*, and *Apoc2* [9]. CREBH also regulates FGF21 [9], which stimulates LPL-mediated TG clearance [95], thereby contributing to hypertriglyceridemia in CREBH KO mice. FGF21 deficiency in ApoE KO mice results in severe atherogenic phenotypes [96], but administering FGF21 to these ApoE KO mice ameliorates atherosclerosis [97]. Thus, further research is required to determine how the molecular mechanism of the progression of atherosclerosis in CREBH KO mouse models contributes to dysfunction of the CREBH-FGF21 pathway. 

## 9. CREBH Rhythmically Interacts with the Transcription Factors for Lipid Metabolism

The proteolytic activation of CREBH in the liver exhibits typical circadian rhythms controlled by the core clock oscillator brain and muscle arnt-like protein-1 (BMAL1) and the AKT/glycogen synthase kinase 3β (GSK3β) signaling pathway. GSK3β-mediated phosphorylation of CREBH modulates the association between CREBH and the coat protein complex II transport vesicles including Sec23, Sec24, and Sar1, and thus—in a circadian manner—controls the ER-to-Golgi transport and subsequent proteolytic cleavage of CREBH [98]. CREBH may indirectly interact with Sec24 through a potential scaffold protein like the SREBP escort protein SCAP. This raises interesting questions about the effects of CREBH on the SREBP cleavage system. 

The circadian clock regulates CREBH proteolytic cleavage and post-translational acetylation modification. Functionally, CREBH is required to maintain circadian homeostasis of hepatic glycogen storage and blood glucose levels. CREBH regulates the rhythmic expression of the genes encoding the rate-limiting enzymes for glycogenolysis and gluconeogenesis, including *Pygl*, *Pck1*, and *G6pc* [38]. CREBH interacts with PPARα to synergize its transcriptional activities in hepatic gluconeogenesis [38]. In regulating hepatic glucose metabolism in mice, acetylation of CREBH at lysine residue 294 controls the CREBH–PPARα interaction and synergy [38]. CREBH deficiency leads to reduced blood glucose levels but increased hepatic glycogen during the daytime period or upon fasting [38]. CREBH has a crucial role to control glucose homeostasis under the circadian clock or metabolic stress. 

CREBH has reciprocal interactions with PPARα and LXRα, as well as the circadian oscillation activator DBP and repressor E4BP4. CREBH regulates and interacts with PPARα [8] or LXRα [98] to enhance CREBH transcriptional activity. CREBH interacts with the circadian oscillation activator DBP and repressor E4BP4 to modulate CREBH transcriptional activity during the night-to-day transition period [98]. The phase of CREBH–DBP interaction is complementary to that of CREBH–E4BP4 interaction, suggesting that DBP and E4BP4 may compete to interact with CREBH and thereby modulate CREBH activities during various circadian phases [98]. PPARα interacts with CREBH in the circadian phase that partially overlaps with the CREBH–LXRα interaction [98]. The interactions among CREBH, PPARα, and LXRα may represent enhancing mechanisms facilitating CREBH peak activity. LXRs consist of two isoforms—LXRα and LXRβ—acting as whole-body cholesterol sensors, and their activation results in a net elimination of cholesterol from the body and amelioration of the plasma lipoprotein profile by mobilization of cholesterol from the periphery [99,100], promoting its excretion and limiting its absorption [101,102,103], reducing its cellular uptake [104], and enhancing its conversion to bile acids in mice [105]. Therefore, CREBH may play a crucial role as a modulator for not only triglyceride metabolism but also cholesterol metabolism in the liver. CREBH may function as a circadian-regulated liver transcriptional regulator integrating energy metabolism and circadian rhythms.

## 10. Conclusions

The regulatory functions of CREBH include gene expression encoding lipogenic regulators, triglyceride synthesis enzymes, enzymes or regulators in lipolysis and lipid transport, fatty acid elongation enzymes, and fatty acid oxidation or cholesterol biosynthesis enzymes to regulate hepatic lipid metabolism. CREBH also acts as a modulator to regulate some transcription factors related to lipid metabolism. In particular and importantly, CREBH interacts with PPARα to regulate the expression of PPARα target genes, including FGF21. PPARα improves systemic lipid metabolism; thus, PPARα agonists can improve hypertriglyceridemia. In the small intestine, CREBH suppresses cholesterol absorption from diet. There is a possibility that CREBH maintains lipid metabolism in the interaction between the liver and the small intestine. CREBH overexpression in mice improves diabetes, obesity, hypertriglyceridemia, and hypercholesterolemia. Conversely, CREBH KO mice exhibit fatty liver and atherosclerosis. CREBH can therefore be a therapeutic target for the treatment of hypertriglyceridemia. Further elucidation of the interaction between CREBH and other transcription factors should increase the importance of CREBH as a regulator for metabolism. An important question remains unanswered as to how CREBH is escorted to Golgi for cleavage and nuclear entry, which could be a good pharmacological target. Therapeutic strategies designed to modulate CREBH activity might be beneficial in the treatment of hyperlipidemia and obesity-associated metabolic diseases.

## Figures and Tables

**Table 1:** The list of cAMP-responsive element-binding protein H (CREBH) direct target genes and mediating co-factors.

Metabolic Function	Target Gene	Co-Factor	Reference
Metabolic hormone	*Fgf21*	PPARα	[8,10]
Gluconeogenesis	*Pck1*, *G6pc*	CRTC2	[11,12]
Fatty acid oxidation	*Ppara*	–	[8,10]
	*Cpt1a*	–	[7]
Ketogenesis	*Bdh1*	–	[7]
Apolipoprotein	*Apoa1*	HNF4α	[13]
	*Apoa4*, *Apoa5*, *Apoc2*, *Apoc3*	–	[9,14]
	*Apob*	–	[15,16]
SREBP suppressor	*Insig2a*	–	[17]
Fatty acid elongation	*Elovl2*, *Elovl5*, *Elovl6*	–	[18]
Lipid droplet formation	*Fsp27β*	–	[19]
Cholesterol absorption	*Npc1l1*	–	[20]

SREBP: sterol regulatory element-binding protein, PPARα: Peroxisome proliferator-activated receptor α, CRTC2: CREB/CREB-regulated transcriptional coactivator 2, HNF4α: hepatocyte nuclear factor 4α, cAMP: cyclic adenosine monophosphate

**Table 2:** The list of transcription factors regulating *Fgf21* expression.

Transcription Factor	Inducer	Reference
**Up-Regulation**		
CREBH	Fasting	[8,10]
PPARα	Fasting, Fibrates	[8,10]
Activating transcription factor 4 (ATF4)	Endoplasmic reticulum (ER) stress, Amino acid deprivation	[31,32,33]
CCAAT enhancer binding protein homologous protein (CHOP)	ER stress	[31]
X-box-binding protein 1 (XBP1)	ER stress, Fasting	[30,34]
Carbohydrate-responsive element-binding protein (ChREBP)	Carbohydrate	[35,36]
**Down-Regulation**		
Liver X receptor (LXR)	Cholesterol	[37]
